# Polo-like kinase 1 overexpression is an early event in the progression of papillary carcinoma

**DOI:** 10.1038/sj.bjc.6601540

**Published:** 2004-01-20

**Authors:** Y Ito, E Miyoshi, N Sasaki, K Kakudo, H Yoshida, C Tomoda, T Uruno, Y Takamura, A Miya, K Kobayashi, F Matsuzuka, N Matsuura, K Kuma, A Miyauchi

**Affiliations:** 1 Kuma Hospital, 8-2-35, Shimoyamate-dori, Chuo-ku, Kobe 650–0011, Japan; 2 Department of Molecular Biochemistry and Clinical Investigation, Osaka University Graduate School of Medicine, Osaka, Japan; 3 Department of Biochemistry, School of Allied Health Science, Osaka University Faculty of Medicine, Osaka, Japan; 4 Department of Pathology, School of Allied Health Science, Osaka University Faculty of Medicine, Osaka, Japan; 5 Department of Pathology, Wakayama Medical School, Wakayama, Japan

**Keywords:** PLK1, immunohistochemistry, thyroid

## Abstract

Polo-like kinase 1 (PLK1) is one of the serine threonine kinases that contributes to cell mitosis and is regarded as a marker of cellular proliferation. However, its protein expression in human carcinoma has not been studied in depth. We investigated PLK1 expression in various thyroid neoplasms in order to elucidate its physiological significance in thyroid carcinoma. Normal follicular cells only occasionally expressed PLK1. In follicular tumours and anaplastic carcinoma, PLK1 overexpression was not a common event and only 5.9% of follicular adenoma, 7.1% of follicular carcinoma, and 11.8% of anaplastic carcinoma overexpressed this protein. However, 43.7% of papillary carcinoma overexpressed PLK1. Polo-like kinase 1 overexpression was more frequently observed in smaller papillary carcinoma lesions, and 62.5% of microcarcinoma (ranging from 4 mm to 1.0 cm) and even 66.7% of incidental carcinoma (less than 4 mm) overexpressed it, whereas this phenomenon could only be seen in 20.0% of lesions larger than 4.0 cm. Furthermore, PLK1 overexpression was not related to cell-proliferating activity evaluated by Ki-67 labelling index, but it was inversely linked to UICC stage, extrathyroidal invasion, and the presence of poorly differentiated lesion as proposed by Sakamoto *et al*. These findings strongly suggest that, unlike other carcinomas previously studied, PLK1 does not act as a cell cycle regulator but plays a constitutive role in papillary carcinoma especially in the early phase, and may contribute to the malignant transformation of this carcinoma.

Thyroid carcinoma is one of the most common malignancies originating in the endocrine glands. There are two prominent histological types of thyroid carcinoma originating from follicular cells, papillary carcinoma and follicular carcinoma, which is comparatively rare. Follicular carcinoma is thought to arise from pre-existing follicular adenoma, although this has not yet been confirmed. The precursor lesion of papillary carcinoma has not yet been identified (Fagin, 2000). Generally, the biological activity of papillary carcinomas is mild, but when these lesions dedifferentiate and turn to anaplastic carcinoma (undifferentiated carcinoma), they grow rapidly and patients usually have a dire prognosis despite various therapeutic strategies (Aldinger *et al*, 1978). These polarised characteristics are typical of thyroid carcinoma, prompting many researchers to investigate factors linked to or triggering dedifferentiation. From a morphological perspective, Sakamoto *et al* (1983) found that papillary or follicular carcinoma with typical growth patterns, such as a solid, trabecular, or scirrhous pattern, shows a poorer prognosis than that with a pure papillary or follicular pattern only. Therefore, they hypothesised that these carcinomas are dedifferentiating, and designated such lesions as poorly differentiated carcinoma, although this hypothesis has not been confirmed.

For the development of neoplasm, the activity of cell cycle progression, including mitosis, is important. To date, expression of various regulators of this event has been investigated in human neoplasms, including thyroid carcinoma. Polo-like kinase 1 (PLK1) is a serine threonine kinase, which belongs to the polo-like kinases (PLKs) family homologous to the *Drosophila polo* kinase (Golsteyn *et al*, 1994; Hamanaka *et al*, 1994). This protein is closely associated with cell cycle regulation. Golsteyn *et al* (1995) showed that, although the level of PLK1 protein expression is low in the G1 phase, it elevates in the S phase following maximal level in the G2/M phase. They also proved that its activity is also elevated during cell mitosis. Using cell lines and human carcinoma tissues, another group also demonstrated that PLK1 is a marker of cell proliferation, which could be useful for carcinoma diagnosis and may be a target for cancer chemotherapy (Holtrich *et al*, 1994; Yuan *et al*, 1997). To date, however, PLK mRNA expression has been investigated for a large number of human carcinoma specimens of only a few origins (Wolf *et al*, 1997; Knecht *et al*, 1999; Tokumitsu *et al*, 1999). In addition, PLK1 protein expression has been studied only for colorectum carcinoma (Takahashi *et al*, 2003), indicating that our knowledge of PLK1 expression and its physiological role in human carcinoma remains limited.

These results prompted us to investigate PLK1 expression in thyroid neoplasms, to elucidate whether this kinase is associated with progression of this carcinoma.

## MATERIALS AND METHODS

### Cell line

A thyroid tumour cell line, NPA (papillary carcinoma), was established by our co-workers. Cells were cultured in Dulbecco's modified Eagle's medium (Nihonseiyaku, Tokyo, Japan) supplemented with 10% fetal calf serum (Dainippon Pharmaceutical, Tokyo, Japan), 500 U ml^−1^ penicillin (Gibco BRL, MD, USA), and 500 *μ*g ml^−1^ streptomycin.

### Tissue specimens

Tissue specimens of thyroid neoplasms were obtained from 149 patients who underwent surgery in the Department of Surgery, Kuma Hospital. This project was approved by the ethics committee of the hospital and informed consent was obtained from the participating patients. Our series involved 17 anaplastic (undifferentiated) carcinomas. These patients underwent surgery between 1987 and 2002. Between 1999 and 2001, 1205 patients underwent surgery for papillary or follicular carcinoma, and 155 (12.9%) had lesions with solid, trabecular, or scirrhous growth patterns diagnosed as poorly differentiated carcinoma, as proposed by Sakamoto *et al* (1983). From these patients, we selected 87 papillary carcinomas and 28 follicular carcinomas (eight widely invasive and 20 minimally invasive carcinomas) for the present study. Of these cases, 28 papillary carcinomas and three follicular carcinomas were diagnosed as including poorly differentiated lesions according to Sakamoto *et al* (1983). Among this series of papillary carcinomas, 36 cases were diagnosed as microcarcinoma according to the WHO classification, because the maximal diameters were 1.0 cm or less (Hedinger *et al*, 1988). Generally, ultrasonography can detect tumours measuring 4 mm or more. In all, 24 of 36 cases were preoperatively diagnosed and surgical treatment was performed for the disease. However, the remaining 12 cases had not been detected by ultrasonography and were incidentally found in specimens following surgery for other thyroid diseases such as follicular adenoma, adenomatous goiter, and diffuse hyperplasia. In these cases, the maximal diameters were 3 mm or less. In addition, we selected 17 follicular adenomas from patients (Hedinger *et al*, 1988) who underwent surgery in 2002. For immunohistochemical study, tissues were fixed in 10% formalin and paraffin-embedded. For Western blot analysis, tissues were snap frozen in liquid nitrogen and stored at −80°C until used.

### Antibody

A mouse monoclonal anti-human PLK1 antibody and anti-Ki-67 antibody were obtained from Transduction Laboratories (Lexington, KY, USA) and Ylem (Rome, Italy), respectively. These were applied at concentrations of 1 : 100 and 1 : 50, respectively.

### Western blot analysis

For Western blotting of PLK1, 20 *μ*g of proteins extracted from tissue specimens was electrophoresed on an 8% polyacrylamide gel. After blotting onto a PVDF membrane, the membrane filter was incubated with 1/1000 diluted anti-human PLK antibody at a concentration of 1 : 200 for 2 h at room temperature. The filter was washed three times with TBS containing 0.05% Tween 20 for 10 min each and then incubated with TBS containing 1/2500 diluted peroxidase-conjugated goat antibody to mouse IgG (Promega, USA) for 1 h. After washing the membrane three times with TBS containing 0.05% Tween 20 for 10 min each, it was developed by an enhanced chemiluminescence system (ECL, Amersham) according to the manufacturer's protocol.

### Immunohistochemistry

Tissue sections 4 *μ*m thick were dewaxed and endogenous peroxidase activity was blocked with 0.3% hydrogen peroxide in methanol for 15 min. For antigen retrieval, sections were immersed in 0.03 mol l^−1^ citrate buffer and incubated at 95°C for 40 min. After rinsing in phosphate-buffered saline pH 7.2 (PBS), 10% bovine serum (Wako, Osaka, Japan) was applied for 20 min to block nonspecific reactions. Then, sections were incubated with a primary antibody at 4°C overnight. After rinsing in PBS, specimens were treated with peroxidase-labelled anti-mouse and anti-rabbit immunoglobulins (Nichirei, Tokyo, Japan) for 30 min. The peroxidase reaction was visualised by incubating the sections with 0.02% 3,3′-diaminobenzidine tetrahydrochloride in 0.05 M Tris buffer with 0.01% hydrogen peroxide (Nichirei, Tokyo, Japan). The sections were counterstained with haematoxylin. Sections for the negative control were prepared using mouse immunoglobulin instead of primary antibody.

### Immunohistochemical evaluation

Polo-like kinase 1 immunoreactivity was observed predominantly in the cytoplasm, and we regarded cells positive for PLK1 only when the immunoreactivity was clearly observed. Based on the positive tumour cell rate, we categorised all cases into four groups: (−), 0–5%; (+), 6–29%; (++), 30–59%; and (+++), more than 60%. Cases graded as (++) or (+++) were regarded as highly overexpressing PLK1, whereas those graded as (−) or (+) were judged as showing low expression of PLK1. This categorisation is fundamentally similar to that in a previous immunohistochemical study using the same antibody (Takahashi *et al*, 2003).

We counted cells positive for Ki-67 by evaluating at least 300 cells in at least three randomly selected fields and calculated the labelling index (LI) for Ki-67. In papillary carcinoma, cases showing Ki-67 LI larger than 3% were classified as the high group.

### Statistical analyses

Fisher's exact probability test was adopted to examine the relationship between variables. A *P*-value less than 0.05 was considered significant.

## RESULTS

### Western blot analysis for PLK1 in thyroid tissues and cell lines

**Figure 1:**
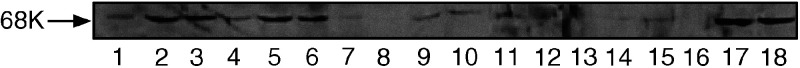
Western blot of PLK1 on thyroid carcinoma tissues and thyroid tumour cell lines. Twenty *μ*g of total cellular proteins were electrophoresed on 12% SDS-PAGE and then Western blot of PLK1 was performed as described in Materials and Methods. Lanes 1–5 indicate papillary carcinoma, lanes 6–10 follicular carcinoma, lanes 11–14 normal thyroid tissue, lanes 15–17 anaplastic carcinoma, and lane 18 thyroid carcinoma cell line, NPA.

We investigated the expression of PLK1 in thyroid carcinomas and cell lines using Western blot analysis (Figure 1

). Lanes 1–5 indicated papillary carcinoma, and lanes 2, 3, and 5 were positive for PLK1. Lanes 6–10 were follicular carcinomas, and lane 6 was the only positive case of PLK. There were no signals observed in normal thyroid tissues (lanes 11–14, Figure 1). Lanes 15–17 indicated anaplastic carcinomas, and a signal for PLK1 as a single band at 67 kDa was observed in one case in lane 17. Polo-like kinase 1 signal was found in a thyroid carcinoma cell line, NPA (lane 18). These results for tissue specimens were coincident with our immunohistochemical findings in each case.

### Immunohistochemistry for PLK1 in thyroid neoplasms

**Figure 2:**
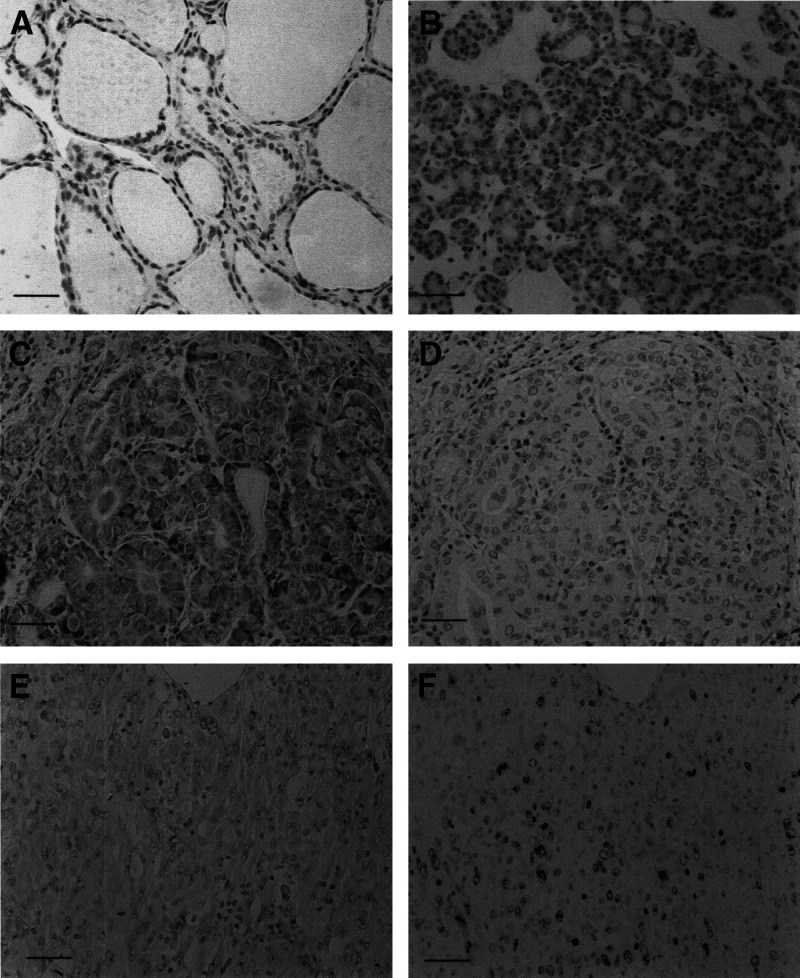
Immunostaining of PLK1 and Ki-67 in thyroid tissues. (**A**) PLK1 was not expressed in normal follicules. (**B**) Absence of PLK1 expression in follicular carcinoma. (**C**) PLK1 overexpression in papillary carcinoma. (**D**) Absence of Ki-67 antigen expression in a serial section with (**C**). (**E**) PLK1 was not overexpressed in anaplastic carcinoma. (**F**) Ki-67 antigen expression is frequently observed in a serial section with (**E**) Scale bar 150 *μ*m.

Polo-like kinase 1 was only occasionally expressed in normal follicular cells, as well as infiltrating lymphocytes (Figure 2A

). Polo-like kinase 1 expression in various types of thyroid neoplasm is summarised in Table 1

**Table 1:** Expression of PLK1 in thyroid neoplasms

	**PLK1 expression**				
	**Low**		**High**		
**Histological type**	**−**	**+**	**++**	**++**	**Total**
Anaplastic caccinoma*	7	8	0	2	17
Papillary carcinoma*,**	26	23	27	11	87
Follicular carcinoma**	21	5	2	0	28
Follicular adenoma	14	2	1	0	17

* *P*=0.0143

** *P*=0.0002.

.

### Follicular carcinoma and adenoma

In follicular tumours, PLK1 overexpression was not frequently observed. Only one of the 17 adenomas (5.9%) and two of the 28 carcinomas (7.1%) were classified in the high group (Figure 2B). There was no significant difference in the incidence of PLK1 overexpression between follicular adenoma and carcinoma.

### Papillary carcinoma

Papillary carcinoma overexpressed PLK1 more frequently than follicular carcinoma (*P*=0.0002), and 38 cases were judged in the high group (43.7%) (Figure 2C). Among the papillary carcinomas in this study, 36 cases were microcarcinoma with maximal diameters of 1.0 cm or less. Of these lesions, 24 were preoperatively diagnosed and their sizes ranged from 4 mm to 1.0 cm. Polo-like kinase 1 overexpression was observed in 15 of these cases (62.5%). Furthermore, the remaining 12 cases were incidental carcinomas, 3 mm in diameter or smaller. Also in these cases, PLK1 was frequently overexpressed and eight cases (66.7%) were regarded in the high group. As shown in Table 2

**Table 2:** Relationship between PLK1 expression in papillary carcinoma and tumour size

	**PLK1 expression**				
	**Low**		**High**		
	**−**	**+**	**++**	**++**	**Total**
⩽0.3 cm^a^^,^^b^	2	2	6	2	12
>0.3 cm, ⩽1.0 cm^b^	8	1	11	4	24
>1.0 cm, <4.0 cm^c^	9	15	10	2	36
>4.0 cm^c^	7	5	0	3	15
					
Total	26	23	27	11	87

a These carcinomas were incidentally found in surgical specimens resected for other thyroid diseases (incidental carcinoma).

b *vs*

c *P*=0.0020.

, larger papillary carcinomas showed a decrease in PLK1 expression (*P*=0.0020), and of 15 tumours larger than 4.0 cm, only three (20.0%) overexpressed PLK1.

We investigated the relationship between PLK1 overexpression and other clinicopathological parameters of papillary carcinoma (Table 3

**Table 3:** Relationship between PLK1 expression and clinicopathlogical parameters other than tumour size of papillary carcinoma excluding incidental carcinoma

	**Low**	**High**	**Total**
Age (years) (mean±s.d.)	56.4±11.4	53.3±14.6	
		NS	
*Gender*			
Male	5	3	8
Female	40	27	67
		NS	
*Lymph node metastasis*			
Absent	19	12	31
Present	26	18	44
		NS	
*Multiple tumour formation*			
Absent	24	20	44
Present	21	10	31
		NS	
*UICC stage*			
I	13	18	31
II	14	5	19
III or IV	18	7	25
	*P*=0.0093 (stage I *vs* stage ⩾II)		
*Extrathyroidal invasion*			
Absent	29	26	55
Present	16	4	20
*Solid, scirrhous, or trabecular growth patterns*			
Absent	21	26	47
Present	24	4	28
*Ki-67 LI*			
Low	35	23	58
High	10	7	17
		NS	
			
Total	45	30	75

). For this analysis, incidental carcinomas were omitted, because most parameters for these cases were unknown. Polo-like kinase overexpression was inversely linked to UICC early stage (*P*=0.0093), extrathyroidal invasion (*P*=0.0372), and the presence of a lesion showing solid, trabecular, or scirrhous growth pattern (*P*=0.0005). Furthermore, we compared PLK1 expression and Ki-67 LI in papillary carcinoma. Generally, cell-proliferating activity of thyroid carcinoma except for anaplastic carcinoma is very low. Also in papillary carcinoma in our series, when we set the cutoff value at 3%, only 17 cases were classified in the high group. As shown in Figure 2C and D, coexpression of PLK1 and Ki-67 was only occasionally seen, and we could not establish any relationship between PLK1 expression and Ki-67 LI in papillary carcinoma (Table 3).

### Anaplastic carcinoma

Anaplastic carcinoma originats from papillary and follicular carcinomas, and has a high activity of development. Indeed, in our series, Ki-67 LI was high and averaged 38.7+16.6 (15.3–60.9). However, in this carcinoma, PLK1 overexpression was seen only in two of the 17 cases (11.8%) (Figure 2E), and the incidence was significantly lower than that in papillary carcinoma (*P*=0.0143). Furthermore, we could not find any relationship between PLK1 expression and Ki-67 antigen expression in this carcinoma (Figure 2E and F).

## DISCUSSION

In this study, we demonstrated that (1) follicular cells in normal follicles only occasionally expressed PLK1, (2) the incidence of PLK1 overexpression was not high in either follicular tumours or anaplastic carcinoma, and (3) in papillary carcinoma, PLK1 overexpression was more frequently observed in cases showing low biologically aggressive phenotypes, although it was not related to cell-proliferating activity.

To date, PLK mRNA expression has been studied in a few types of carcinoma such as non-small cell lung carcinoma, squamous cell carcinoma of the head and neck, gastric carcinoma, and oesophageal carcinoma (Wolf *et al*, 1997; Knecht *et al*, 1999; Tokumitsu *et al*, 1999). Except for that in gastric carcinoma, the PLK mRNA level in these carcinomas was directly linked to the biologically aggressive phenotype of the tumour and/or worse prognosis for the patient (Wolf *et al*, 1997; Knecht *et al*, 1999; Tokumitsu *et al*, 1999). Furthermore, Takahashi *et al* (2003) demonstrated that, in human colorectal carcinoma, PLK1 protein overexpression was directly related to the proliferating cell nuclear antigen (PCNA) LI, as well as some other parameters reflecting the biological aggressiveness of the tumour such as Dukes' classification, primary tumour invasion, lymph node metastasis, and aberrant p53 expression. These results are not discrepant with and even supported by those obtained from previous *in vitro* studies that PLK1 plays a significant role in cell-proliferating activity (Holtrich *et al*, 1994; Golsteyn *et al*, 1995; Yuan *et al*, 1997).

However, our findings are sharply in contrast with those in other carcinomas, and the physiological significance of PLK1 in thyroid tumours seems complicated. A high incidence of PLK1 overexpression was observed in papillary carcinoma. As previously reported, cell-proliferating activity of papillary carcinoma is generally low (Erickson *et al*, 1998), and in our series, Ki-67 LI was less than 3% in most cases. We investigated the relationship between PLK1 overexpression and cell-proliferating activity of papillary carcinoma, but there was no association detected, indicating that PLK1 does not play a role in regulating cell proliferation. Instead, we found that PLK1 is more frequently overexpressed in papillary carcinoma with low biological aggressiveness, because its expression was inversely linked to certain clinicopathological parameters such as tumour size, UICC stage, extrathyroidal invasion, and the presence of poorly differentiated lesion. Especially, it is interesting to that PLK1 is more frequently overexpressed in papillary microcarcinoma and even in incidental carcinoma, which is smaller than 4 mm in maximal diameter. Recently, we performed a trial of observation without surgical treatment for papillary microcarcinoma patients, and found that most of these tumours do not enlarge, indicating that these lesions do not grow or grow only slowly (Ito *et al*, 2003). These findings strongly suggest that PLK1 is not related to growth of papillary carcinoma. Another interesting finding is the frequent lack of PLK1 expression in anaplastic carcinoma. Anaplastic carcinoma is known to arise from papillary and follicular carcinomas and has high mitotic activity as demonstrated by Erickson *et al* (1998) as well as this study. However, in our series, PLK1 overexpression was observed only in 11.8% of cases. According to these findings, we can conclude that PLK1 is not likely to contribute directly to the mitosis of papillary carcinoma cells. This means that the physiological significance of PLK1 in thyroid papillary carcinoma is unique and completely different from that in all other carcinomas studied to date. One explanation of these results is that PLK1 plays an oncogenic role and is constitutively required in papillary carcinoma during the early phase, while it is less necessary for the development of this carcinoma during the advanced stage. Although the mechanism of malignant transformation from normal follicle to papillary carcinoma remains to be elucidated, our finding that PLK1 is frequently overexpressed in incidental carcinomas suggests that PLK1 expression may even play a role in the carcinogenesis of papillary carcinoma.

In follicular tumours, PLK1 overexpression was not frequently detected, and there was no significant difference in the incidence between follicular carcinoma and follicular adenoma. It is therefore suggested that this protein is not likely to play a role in the progression of this tumour or in the transformation of follicular adenoma to carcinoma.

In summary, the findings of this study strongly suggest that PLK1 plays a constitutive role in thyroid papillary carcinoma especially during the early phase, and may be related to the malignant transformation of this lesion. Further studies are necessary to elucidate the detailed mechanism by which this protein contributes to carcinogenesis and the development of this carcinoma.
